# Primary hypertrophic osteoarthropathy and bilateral transient lateral patellar dislocation in an adolescent

**DOI:** 10.1259/bjrcr.20210121

**Published:** 2021-12-24

**Authors:** Sharenja Jeyabaladevan, Emmanouil Astrinakis, Margaret Callan, Paul Anthony Sookur

**Affiliations:** 1 Department of Clinical Imaging, Chelsea and Westminster Hospital NHS Foundation Trust, London, United Kingdom; 2 Department of Rheumatology, Chelsea and Westminster Hospital NHS Foundation Trust, London, United Kingdom

## Abstract

This case report is of the index case of bilateral transient patellar dislocation in a patient with primary hypertrophic osteoarthropathy. Primary hypertrophic osteoarthropathy is a rare complex disorder with variable presentation and thus frequently delayed diagnosis. Notably, this disease has multiple skeletal manifestations and, of relevance to this case, a proportion of patients suffer from osteitis. Our patient had serial imaging of the knee joints demonstrating osteitis and associated alteration of the femoral trochlea morphology, predisposing to bilateral transient lateral patellar dislocation. The patient’s clinical presentation, diagnosis and management are discussed. Classification of the disease and its diagnostic parameters are summarised along with key imaging features amongst various imaging modalities.

## Introduction

Primary hypertrophic osteoarthropathy (PHOA), also known as pachydermoperiostosis, is a rare monogenic disorder, characterised by aberrant proliferation of skin and periosteal tissue and often presenting in childhood or adolescence with periostosis of tubular bones, pachyderma and digital clubbing.^1^

In contrast, secondary hypertrophic osteoarthropathy is more common, usually presents in adulthood and can be due to a multitude of extraskeletal causes, most commonly pulmonary in aetiology.

The definitive mechanism underlying the pathophysiology of PHOA is unclear.^1^ It has been reported to be a disease which is almost exclusively present in males with either autosomal recessive or dominant inheritance.^2^ The inheritance pattern is varied; cases have been described of patients who have the autosomal dominant form with incomplete penetrance, autosomal recessive form and in the offspring of consanguineous couples without prior relevant family history.^3^

It often presents in adolescence with thickening of the skin (pachyderma), particularly involving the face. At least two of the following are required for diagnosis; positive family history, digital clubbing, pachyderma or periostosis on clinical imaging.^4^ The diagnostic features have been split into major and minor criteria.^5,6^ Major criteria include periostosis, pachyderma and finger clubbing. Minor criteria include hyperhidrosis, arthralgia, gastric ulcer, cutis verticis gyrata, blepharoptosis, joint effusion, edema, seborrhea, acne and flushing.^7^

Transient lateral patellar dislocation is often secondary to a dysplastic femoral trochlea. Dysplasia describes flattening of the trochlea resulting in a shallow groove at the anterior femoral cortex. This leads to incomplete tracking of the patella during flexion which predisposes to transient patella dislocation.^8^

## Case report

A 15-year-old male, born in Afghanistan to consanguineous parents (who are first cousins) presented to the dermatology clinic. The patient’s primary concern was regarding thickening of his facial features for which he subsequently had plastic surgery. Over the next few years, he developed pain and swelling particularly affecting his knees and ankles, with large bilateral knee joint effusions, and was referred to the rheumatology team. Etorocoxib 60 mgs provided symptomatic relief. His blood tests demonstrated an elevated ESR (70 mm/hr) and CRP (22 mg l^−1^). In addition, the patient had allergic blepharo-conjuctivitis, acne which had been previously treated with isotretinoin, iron deficiency anaemia treated with IV Ferinject infusion (1g over 30 mins), palmoplantar hyperhidrosis, hyperplastic gastric polyps, all of which are minor features of primary hypertrophic osteoarthropathy. Genetic testing revealed that our patient had a mutation of the SLCO2A1 gene, consistent with a diagnosis of PHOA.

**Figure 1.**
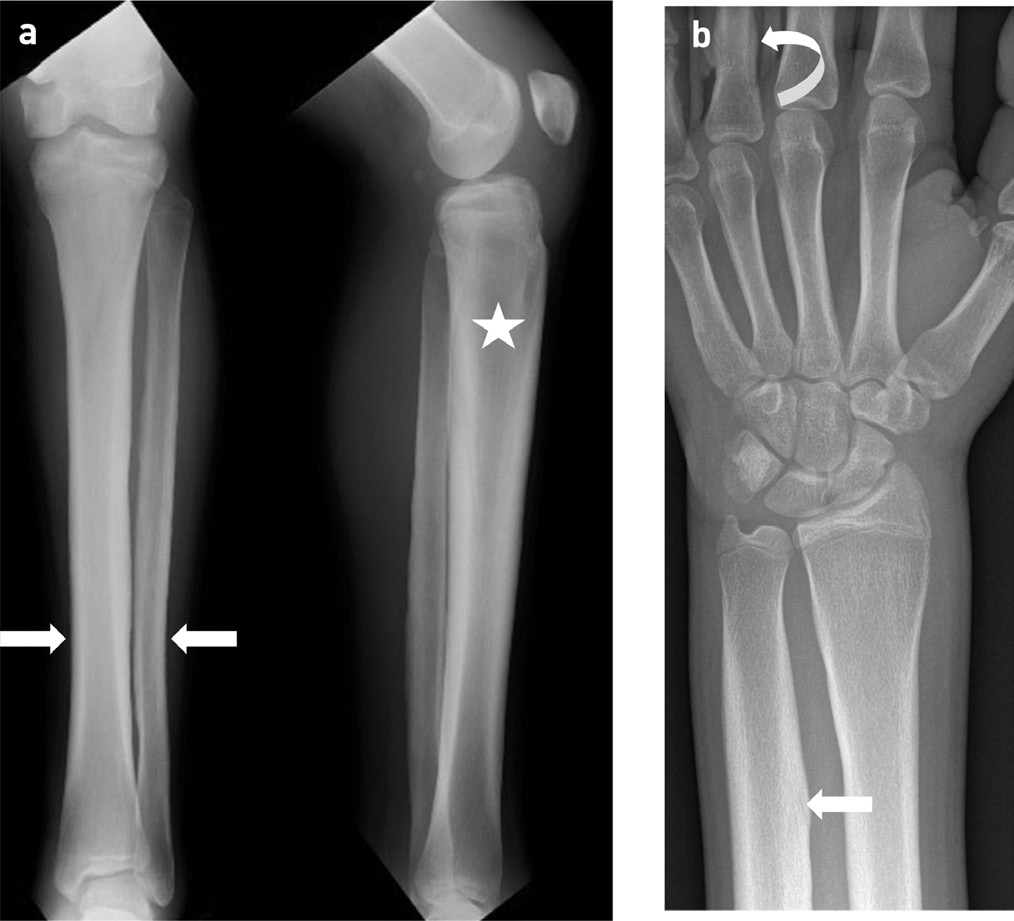
12-year-old boy with primary hypertrophic osteoarthropathy. (**a**) AP and lateral radiographs of the left lower leg demonstrating diffuse lamellar periosteal reaction and cortical thickening of the tibia and fibula (arrows) with enlargement of the bone (star). The appearances of the contralateral leg were identical (not shown). (**b**) AP radiograph of the right wrist demonstrating diffuse cortical thickening of the radius and ulna (arrow) with enlargement of the bones. Further cortical thickening in the proximal phalanges of the fingers (curved arrow) is seen. The changes were symmetrical with identical appearances in the contralateral upper limb (not shown).

**Figure 2.**
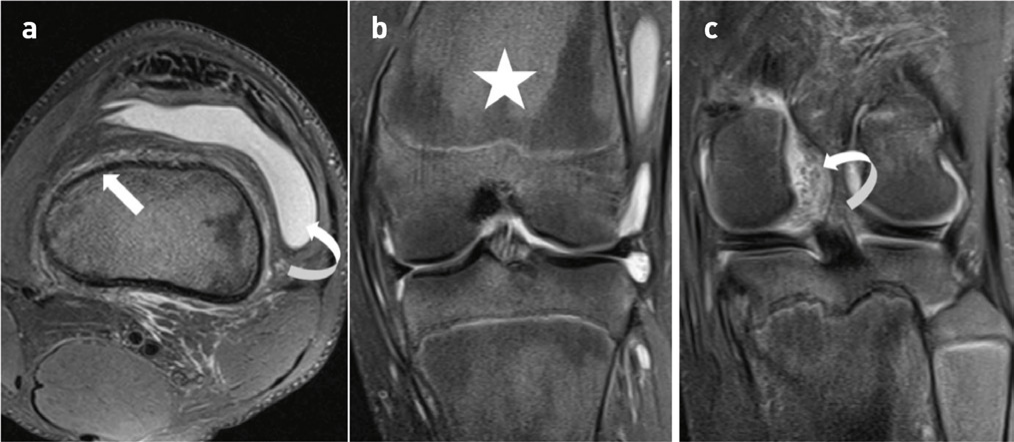
12-year-old boy with primary hypertrophic osteoarthropathy. (**a**) Axial fat saturated proton-density weighted (FSPDW) image of the left knee demonstrating circumferential periostitis in the distal femur (arrow) and a joint effusion (curved arrow). (**b, c**) Coronal FSPDW images of the left knee demonstrating enlargement of the distal femur (star) and evidence of synovial hypertrophy and joint effusion (curved arrow).

**Figure 3.**
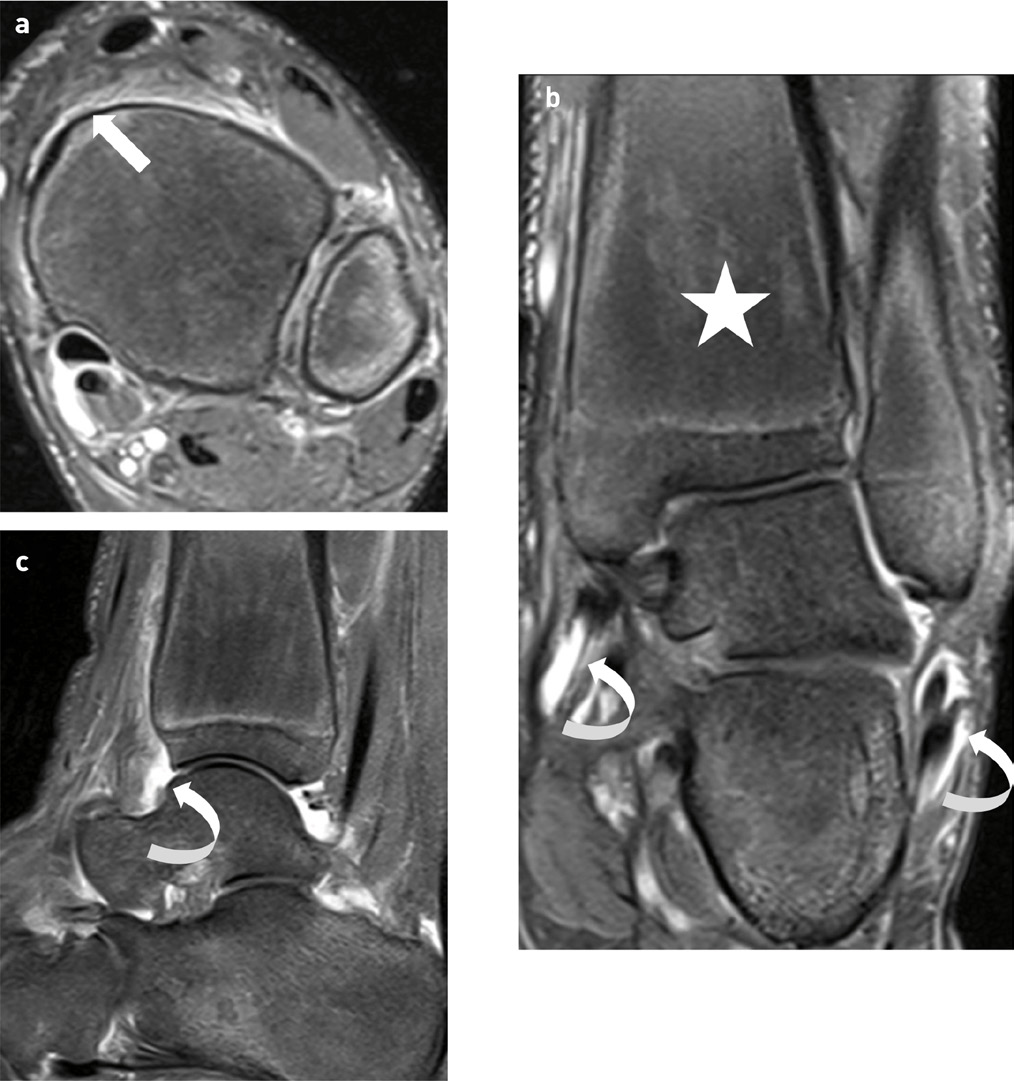
12-year-old with primary hypertrophic osteoarthropathy. (**a**) Axial fat saturated proton density weighted (FSPDW) image of the left ankle demonstrating diffuse periostitis (arrow). (**b**) Coronal FSPDW image demonstrating enlargement of the distal tibia (star). (**c**) Sagittal FSPDW image demonstrating an ankle joint effusion (curved arrow)

Initial plain film imaging from when the patient was 12 years old demonstrated diffuse lamellar periosteal reaction and cortical thickening of the tibia, fibula, distal ulna, distal radius and phalanges (Figure 1) with enlargement of the bone. Additional radiographs highlighted that the periostosis was symmetrical and diffuse. Subsequent MRI of the left knee and ankle (Figures 2 and 3) demonstrated circumferential periostosis, enlargement of the distal femur and distal tibia, synovial hypertrophy, and joint effusions. The patient was frequently troubled by bilateral knee joint effusions which were aspirated (with removal of up to 200 mls synovial fluid) followed by intraarticular injections of 60 mg depomedrone for symptomatic relief under the care of the rheumatologists. As a result serial knee MRIs were obtained and demonstrated the progression of trochlea dysplasia.

**Figure 4.**
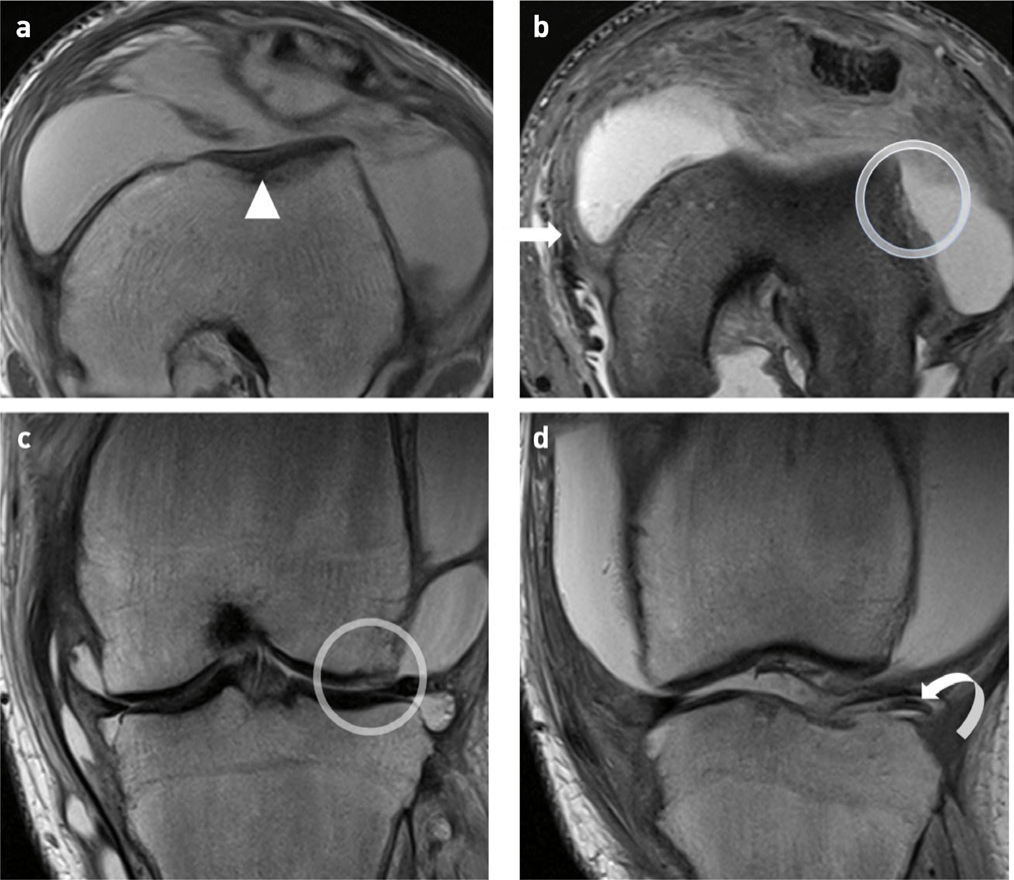
15-year-old boy with primary hypertrophic osteoarthropathy and transient lateral patellar dislocation of the left knee. (**a**) Axial proton density weighted (PDW) image of the left knee demonstrating a shallow trochlear groove (arrowhead). (**b**) Axial fat-saturated proton density weighted image showing evidence of transient lateral patellar dislocation with increased signal in the medial patellofemoral ligament in keeping with a tear (arrow) and a shearing osteochondral injury in the anterolateral aspect of the lateral femoral condyle (circle). (**c, d**) Coronal PDW images showing a defect in the cartilage and subchondral bone plate at the site of the osteochondral injury (circle) and an anteroinferiorly displaced osteochondral fragment (curved arrow).

**Figure 5.**
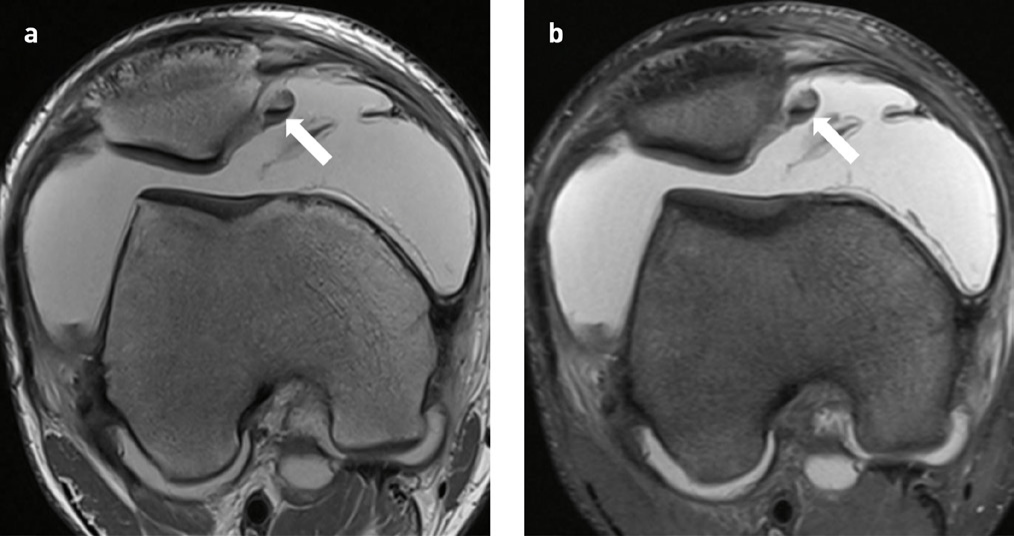
16-year-old boy with primary hypertrophic osteoarthropathy and transient lateral patellar dislocation of the right knee. (**a,b**) Axial proton density weighted (PDW) and axial fat-saturated PDW images of the right knee demonstrating a mildly displaced osteochondral fracture in the medial facet of the patella (arrow).

**Figure 6.**
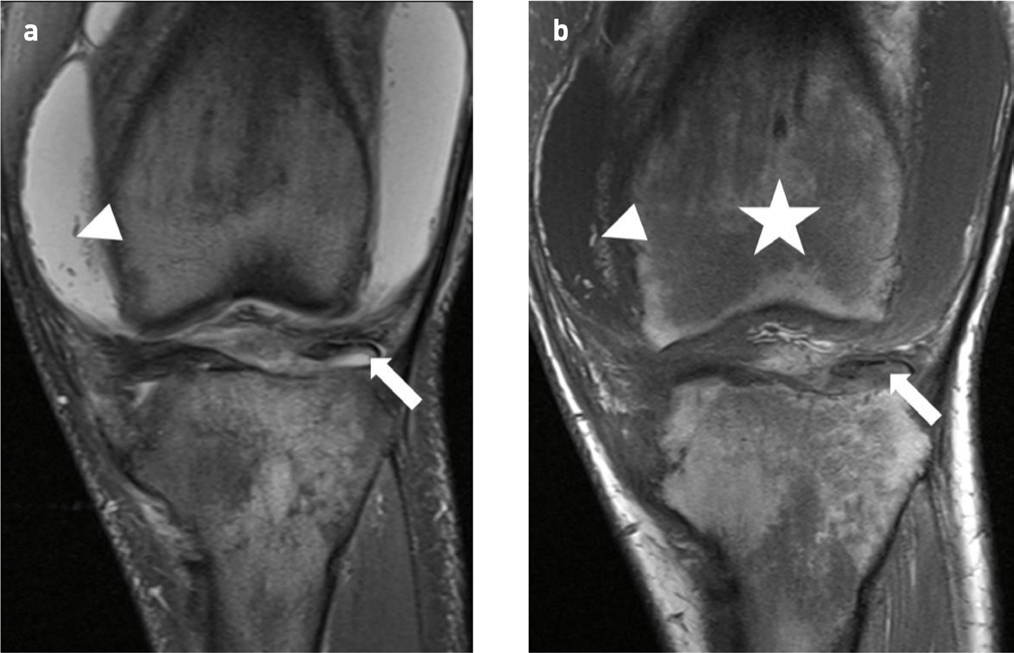
16-year-old boy with primary hypertrophic osteoarthropathy and transient lateral patellar dislocation of the left knee. (**a,b**) Coronal fat saturated proton density weighted (FS PDW) and coronal *T*_1_-weighted (T1W) images of the left knee demonstrating ongoing synovial hypertrophy with fatty metaplasia (arrowheads) and a large joint effusion. Diffusely abnormal bone marrow signal is shown in the metaphyses and epiphyses with loss of the normal fat signal on T1W imaging (star) and hyperintensity on FS PDW imaging due to osteitis. The chronic-displaced osteochondral fragment is seen (arrow).

At the ages of 15 and 16, the patient presented following twisting injuries to both knees on separate occasions whilst playing football. MRI imaging of both knees demonstrated widening of the distal metaphyses resulting in a shallow trochlear groove (Figure 4). The MRI of the left knee following the initial injury illustrated an impaction fracture in the anterolateral aspect of the lateral femoral condyle with a displaced osteochondral fragment and a torn medial patellofemoral ligament (Figure 4). The MRI of the right knee following a subsequent football injury illustrated an osteochondral injury(Figure 5) in the medial facet of the patella with a displaced osteochondral fragment, an impaction injury in the anterolateral aspect of the lateral femoral condyle and a torn MPFL (Figure 6). This pattern of injury is consistent with bilateral transient lateral patellar dislocation.

## Discussion

PHOA is a rare disorder, primarily affecting males with an early onset during childhood or adolescence^5^ and often presents a diagnostic challenge. Radiological findings are key in pointing to the possible diagnosis, and this may then be confirmed by genetic testing.^1^

The pathogenesis of development of periostosis in PHOA is still poorly understood but may involve humoral and/or neurogenic pathways.

The humoral pathway model suggests that aberrant levels of growth factors and cytokines including prostaglandin are responsible for the periostosis in PHOA. This is supported by the observation that neonates with congenital heart disease who are given prostaglandin infusions develop symmetrical periostosis along the diaphyses of long bones similar to that seen PHOA. There is subsequent resolution of the periostosis after the infusion has been stopped.^9^

The neurogenic pathway model suggests that an abnormal neural reflex involving the vagus nerve causes vasodilatation and increased blood flow to the extremities and that this results in diffuse periostosis of the long bones.^10^

Our patient had a mutation involving the SLCO2A1 gene, which encodes a protein responsible for the degradation of prostaglandin. This leads to abnormally high levels of prostaglandin which also causes high levels of vascular endothelial growth factor. Elevated levels of both these factors influence osteoblasts leading to periostosis^1^

Frequently PHOA patients present with pain resulting in plain radiographs of the extremities being obtained. Radiographs will demonstrate periostosis, the imaging hallmark of the disease, along the shaft of tubular bones with widening of the metaphyses as demonstrated in our case. This radiographic finding has been described as present in 80–97% of patients.^11^ Periostosis distribution is often symmetric and diffuse.^1^

Fluid-sensitive MR sequences will be able to demonstrate the elevated periosteum whereby adjacent to the bone there will be hypointense fine lines and surrounding high signal.^12^ MRI imaging is also useful for demonstrating paraosseous soft tissue oedema secondary to the periostosis.^12^ The MRI findings in our patient demonstrated joint effusions and synovial hypertrophy, this is one of the minor features in diagnosis of primary hypertrophic osteoarthropathy. This is less common and only present in 40% of diagnosed patients.^13^ The progression of overactive osteoblastic activity resulted in a shallow trochlear groove which predisposed our patient to bilateral lateral patellar dislocation.

Patellar instability is often linked to syndromes, where joint laxity is cited as the cause and not trochlear dysplasia. Femoral trochlear dysplasia resulting in bilateral patellar dislocation, although rare, has been described as having a genetic association. There have been a few familial case reports of bilateral trochlear dysplasia and recurrent patellar dislocation whereby the mode of inheritance is thought to be autosomal dominant.^14^ Given that our patient had consanguineous parents a genetic cause should be considered for the aetiology of the patient’s trochlear dysplasia.

The Dejour classification of trochlear dysplasia uses a combination of lateral radiographs and axial CT and MR imaging to classify abnormal trochlear morphology. The classification identifies four different types. Type A refers to the presence of a “crossing sign” on a lateral radiograph, the crossing sign is the intersection of the deepest point of the trochlear sulcus with anterior border of the femoral condyles. This in turn denotes the presence of normal facet angles but a shallow trochlea. Type B is the presence of a trochlear spur in addition to the crossing sign. A trochlear spur is the angular anterior projection of the most proximal aspect of the trochlea, this is indicative of a flattened trochlear groove. Type C is the presence of a double contour in addition to the crossing sign, the double contour is best demonstrated on a lateral radiograph, this signifies a hypoplastic medial facet. Type D is best appreciated on axial imaging and it is the presence of a cliff pattern in between the facets, this is in addition to all the signs seen in Types A–C.^14^ Our case demonstrates over the course of several years both the patient’s femoral trochleas became progressively shallow and flat secondary to new bone formation (Figure 4). This then led to bilateral transient patellar dislocation. We believe this to be the first case in the literature which describes transient lateral patellar dislocation in the setting of primary hypertrophic osteoarthropathy. Patients will often have radiographs initially during their presentation, and it is paramount that there is an awareness of the potential causes of multifocal periostosis. Multiple diseases including, thyroid acropachy, and lymphoma can cause multifocal periostosis alongside primary hypertrophic osteoarthropathy.^1^

**Figure 7.**
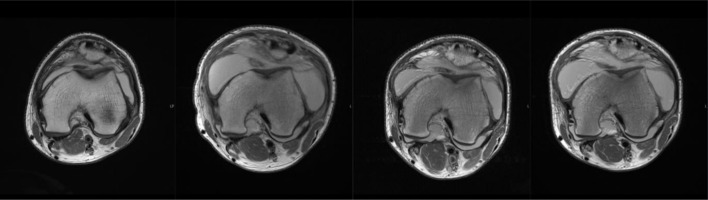
Serial imaging of left knee demonstrating progressive flattening of the trochlear groove (2016, 2017, 2019, 2020 left to right)

**Figure 8.**
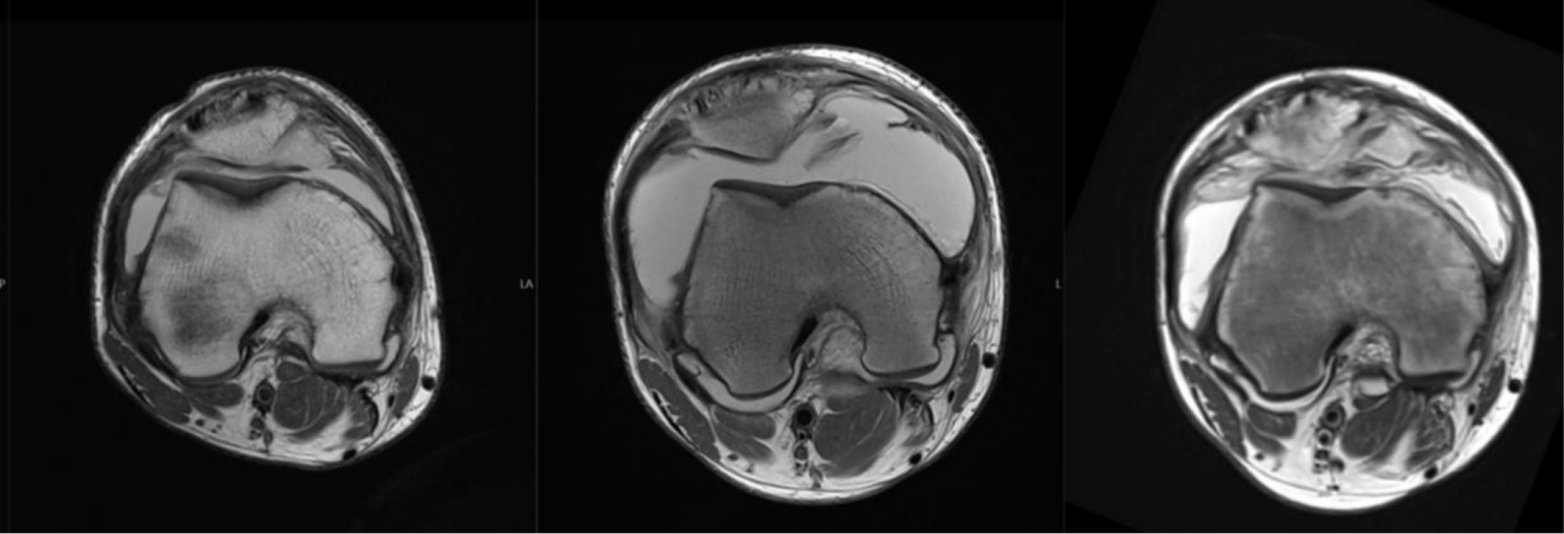
Serial imaging of right knee demonstrating progressive flattening of the trochlear groove (2016, 2018, 2021 left to right)

Our patient had PHOA and progressive alteration of the morphology of the femoral trochlea (Figures 7 and 8) and, to our knowledge, this is the first case in the literature.

## Learning points

- Understand the potential pathophysiological mechanisms causing PHOA.
- Imaging features of PHOA on plain film radiography that would be most useful for the general radiologist.
- Imaging features of PHOA on MRI that would be most useful for a radiologist with a subspecialty interest in MSK.
- Unique to this case, the patient has morphological changes of the femoral trochlea bilaterally and PHOA that has predisposed to patellar dislocation.
